# Covered Metal Stenting for Malignant Lower Biliary Stricture with Pancreatic Duct Obstruction: Is Endoscopic Sphincterotomy Needed?

**DOI:** 10.1155/2013/375613

**Published:** 2013-11-12

**Authors:** Kazunari Nakahara, Chiaki Okuse, Keigo Suetani, Yosuke Michikawa, Shinjiro Kobayashi, Takehito Otsubo, Fumio Itoh

**Affiliations:** ^1^Department of Gastroenterology and Hepatology, St. Marianna University School of Medicine, 2-16-1 Sugao, Miyamae-ku, Kawasaki 216 8511, Japan; ^2^Department of Gastroenterological and General Surgery, St. Marianna University School of Medicine, Kawasaki 216 8511, Japan

## Abstract

*Aims*. To evaluate the need for endoscopic sphincterotomy (EST) before covered self-expandable metal stent (CSEMS) deployment for malignant lower biliary stricture with pancreatic duct obstruction. *Methods*. This study included 79 patients who underwent CSEMS deployment for unresectable malignant lower biliary stricture with pancreatic duct obstruction. Treatment outcomes and complications were compared between 38 patients with EST before CSEMS deployment (EST group) and 41 without EST (non-EST group). *Results*. The technical success rates were 100% in both the EST and the non-EST group. The incidence of pancreatitis was 2.6% in the EST, and 2.4% in the non-EST group (*P* = 0.51). The incidences of overall complications were 18.4% and 14.6%, respectively, (*P* = 0.65). Within the non-EST groups, the incidence of pancreatitis was 0% in patients with fully covered stent deployment and 3.6% in those with partially covered stent deployment (*P* = 0.69). In the multivariate analysis, younger age (*P* = 0.003, OR 12) and nonpancreatic cancer (*P* = 0.001, OR 24) were significant risk factors for overall complications after CSEMS deployment. EST was not identified as a risk factor. *Conclusions*. EST did not reduce the incidence of pancreatitis after CSEMS deployment in patients of unresectable distal malignant obstruction with pancreatic duct obstruction.

## 1. Introduction

In recent years, covered self-expandable metal stent (CSEMS) deployment is widely used for the management of unresectable malignant lower biliary strictures [1–5]. However, physical compression to the pancreatic duct orifice after CSEMS deployment may obstruct the outflow of pancreatic juice and induce pancreatitis [6]. In particular, in cases without tumor invasion to the pancreatic duct, CSEMS deployment was associated with a high incidence of pancreatitis [5, 7, 8]. Thus, in order to ensure drainage of pancreatic juice to prevent pancreatitis, endoscopic sphincterotomy (EST) is often performed before CSEMS deployment [9–11]. However, the need for EST is not clear when the pancreatic duct is already obstructed due to malignant invasion. In pancreatic cancer with obstruction of the pancreatic duct, some articles reported that the incidence of pancreatitis was low [5, 8].

Recently, there have been a few reports describing EST before CSEMS deployment for pancreatitis prevention as being unnecessary when the pancreatic duct is obstructed [8, 12–15]. Furthermore, Artifon et al. [12] reported that EST is associated with higher rates of complications including stent migration, bleeding, and perforation. However, because only a few kinds of the partially covered type CSEMSs were used in these studies, it is not clear whether EST is needed before CSEMS deployment when employing the fully covered type CSEMSs or other partially covered types.

In the present study, we retrospectively assessed the need for EST before deployment of CSEMS including fully covered type stents for malignant lower biliary stricture with pancreatic duct obstruction.

## 2. Materials and Methods

### 2.1. Patients

We retrospectively analyzed the medical records of 2078 patients, who underwent endoscopic retrograde cholangiopancreatography (ERCP) at St. Marianna University School of Medicine Hospital between January 2007 and December 2012. Among them, this study included 79 consecutive patients (40 male and 39 female) who underwent CSEMS deployment for unresectable malignant lower biliary stricture with pancreatic duct obstruction due to tumor invasion. Pancreatic duct obstruction was radiologically defined by abdominal computed tomography (CT), magnetic resonance cholangiopancreatography (MRCP), and/or ERCP when the main pancreatic duct was obstructed by the tumor and also the distal main pancreatic duct was dilated to 4 mm or more. We chose 4 mm as the cutoff since that is at least twice the size of normal pancreatic duct. Lower biliary stricture was defined as those of more than 2 cm downstream from bifurcation of the common bile duct. We excluded patients who underwent CSEMS deployment above the papilla. We also excluded patients with postoperative anatomy such as Billroth II or Roux-en-Y. The etiology of lower biliary stricture was pancreatic cancer in 73 patients, ampullary carcinoma in 4, gallbladder cancer with direct infiltration of pancreas in 1, and pancreatic metastasis from malignant schwannoma in 1. The diagnosis of unresectable malignant biliary obstruction was based on imaging and/or pathological findings before CSEMS deployment. The mean age of the patients was 72.7 years (range: 44–87 years).

The EST group included 38 patients who underwent EST before CSEMS deployment, and the non-EST group included 41 who did not undergo EST. EST was performed using the electrosurgical generator with 120 Watt Endocut mode, and the incision range was small (incision reaching the hooding fold) or medium (incision up to a portion of the hooding fold) in all patients. Although it was difficult to solve the details for performing EST because of the property of retrospective study, many of the reasons for having performed EST were to prevent pancreatitis or to allow easier passage of accessories. Ultimately, the decision of with or without EST was left to the operator's discretion. All ERCPs were performed by several experienced endoscopists.

While all CSEMSs were 10 mm in diameter, fully covered type stents were deployed in 22 patients and partially covered type stents in 57. The fully covered type stents used were WallFlex Biliary RX stents (Microvasive, Boston Scientific Corp.) in 11 patients, Niti-S ComVi stents (Taewoong Medical Inc., Seoul, Korea) in 7, Hanaro stents (MItech, Seoul, Korea) in 3, and Zeostent (Zeon Medical Inc., Tokyo, Japan) in 1. The partially covered type stents used were WallFlex Biliary RX stents (Microvasive, Boston Scientific Corp.) in 33 patients, Niti-S ComVi stents (Taewoong Medical Inc., Seoul, Korea) in 22, and WALLSTENT (Microvasive, Boston Scientific Corp., Natick, MA) in 2. All stents whether fully covered type or partially covered type were positioned across the bile duct orifice with about 5 mm of the distal end of the stent protruding into the duodenum on the stent full expansion.

### 2.2. Measurements

Age, sex, primary diseases, stent types, procedure time, duration of follow-up, technical success rates, dilation rates of stents at the papilla, serum amylase levels, incidence of pancreatitis, and the incidence of complications were retrospectively compared between the EST (*n* = 38) and non-EST (*n* = 41) groups. In addition, serum amylase levels, the incidence of pancreatitis, and the incidence of complications were compared between patients undergoing deployment of a fully covered type stent (FC group, *n* = 13) and those undergoing deployment of a partially covered type stent (PC group, *n* = 28) in the non-EST group. To clarify the necessity of EST, the following variables were assessed by univariate analysis to identify the risk factors for pancreatitis and all complications: gender (female), age (<60 years), etiology of biliary obstruction (nonpancreatic cancer), periampullary diverticulum, non-EST, type of CSEMS (fully covered type), pancreatic duct injections, biopsy of the bile duct, cytology of bile juice, intraductal ultrasonography, and procedure time (>45 minutes). No patient had a sphincter of Oddi dysfunction or history of pancreatitis, so we did not incorporate these factors. We included significant variables (*P* < 0.05) into a multivariate regression analysis.

Technical success was defined as a serum total bilirubin level decrease to at most 3 mg/dL or at most 50% of the previous level within 2 weeks after CSEMS deployment. Based on radiography performed at least 5 days after CSEMS deployment, insufficient dilation was defined as the presence of notch constriction at the site of stent across the papilla, and favorable dilation was defined as the absence of such constriction. Serum amylase levels (normal range: 37–124 IU/L) were measured before 3 hours, 1 day, and 2 days after CSEMS deployment. Diagnoses and severities of complications, such as pancreatitis, bleeding, perforation, and cholangitis, were determined according to the consensus criteria proposed by Cotton et al. [16]. The consensus criteria of pancreatitis were as follows: new or worsening abdominal pain persisting for at least 24 h and requiring analgesics after CSEMS placement in conjunction with an elevation in serum amylase or lipase levels greater than three times the normal upper limit. The grading of severity was as follows: mild, requiring prolongation of planned admission for 3 days or less; moderate, requiring 4–10 days of hospitalization; severe, requiring more than 10 days of hospitalization, intensive care, or surgical intervention. All of the patients who developed pancreatitis underwent CT examination to confirm radiologic evidence of pancreatitis.

### 2.3. Statistical Analysis

Statistical analysis was carried out using the Prism 5 program (GraphPad Software, Inc., CA, USA) and SPSS (version 19; SPSS, Chicago, IL, USA). The *χ* ^2^ test, Fisher's exact test, and Welch's *t*-test were used for statistical analysis where appropriate. As for risk factors for pancreatitis and complications, variables found to be possibly significant (*P* < 0.05) by univariate analysis were chosen for entry into a multiple logistic regression. A *P* value less than 0.05 was considered to indicate a statistically significant difference.

## 3. Results

### 3.1. Comparison between the EST and the Non-EST Groups

There were no differences in age, sex, primary disease, stent types, or duration of the follow-up period between the EST and the non-EST groups. Total procedure times were longer for patients in EST group (34 ± 16 min) as compared with those in the non-EST group (27 ± 12 min) (*P* = 0.03) (Table 1).

The technical success rates were 100% in both the EST and the non-EST groups. Favorable drainage was thus achieved regardless of whether EST was performed.

The rates of stent dilation at the papilla were 100% in both the EST and the non-EST groups. Even without EST, no notch constriction persisted at the stenting site across the papilla in any patient.

As for the mean serum amylase levels, there were no apparent differences between the EST and the non-EST groups (Table 2).

The incidence of pancreatitis was 2.6% (1/38) in the EST group and 2.4% (1/41) in the non-EST group (*P* = 0.51). The pancreatitis was mild in both patients.The primary disease in both of these patients was pancreatic cancer. Two patients developing mild pancreatitis had no risk factors of post-ERCP pancreatitis, such as a suspected sphincter of Oddi dysfunction, history of post-ERCP pancreatitis, pancreatic duct injection, endoscopic papillary balloon dilation, precutting, or pancreatic sphincterotomy. Conservative therapy without stent removal resulted in improvement in each patient.

The overall incidence of complications including pancreatitis was 18.4% (7/38) in the EST group and 14.6% (6/41) in the non-EST group (*P* = 0.65). The complications developed in the EST group were cholecystitis in 2 patients and pancreatitis, bleeding, cholangitis, distal migration, and liver abscess in 1 patient each. Those in the non-EST group were distal migration in 3 patients, cholecystitis in 2, and pancreatitis in 1 (Table 2).

### 3.2. Comparison between the FC and PC Groups within the Non-EST Group

For the mean serum amylase levels, there were no apparent differences between the FC and PC groups. Pancreatitis occurred in only 1 PC group patient. The incidence of pancreatitis was 0% (0/13) in the FC group and 3.6% (1/28) in the PC group (*P* = 0.69) (Table 3).

The overall incidence of complications including pancreatitis was 23.1% (3/13) in the FC group and 10.7% (3/28) in the PC group (*P* = 0.57). The complications developed in the FC group were cholecystitis in 2 patients and distal migration in 1, and those in the PC group were distal migration in 2 patients and pancreatitis in 1.Although the difference was not statistically significant, the incidence of cholecystitis tended to be higher in the FC group (*P* = 0.18) (Table 4).

### 3.3. The Risk Factors for Complications after CSEMS Deployment

Since there were only 2 patients with post-ERCP pancreatitis, it was a too small number of patients to analyze the risk factor of pancreatitis statistically by uni- and multivariate analysis. As for the risk factor of overall complications, age (<60 years) (*P* = 0.022, OR 6.9, CI 1.5, 33) and nonpancreatic cancer (*P* = 0.006, OR 14, CI 2.3, 89) were significant risk factors in the univariate analysis (Table 4). In the multivariate logistic regression analysis, age (<60 years) (*P* = 0.003, OR 12, CI 2.3, 63) and nonpancreatic cancer (*P* = 0.001, OR 24, CI 3.5, 165) were also significant risk factors for the development of complications after CSEMS deployment (Table 5). On the other hand, EST was not identified as a risk factor. The diagnosis of nonpancreatic cancer developing complication was ampullary carcinoma in 3 patients and gallbladder cancer with direct infiltration of pancreas in 1. Three patients with ampullary carcinoma developed distal stent migration. One patient with gallbladder cancer developed liver abscess.

## 4. Discussion

CSEMS deployment has been associated with relatively high incidence of pancreatitis with reported incidences ranging from 0 to 7.3% [2–4, 8, 10, 17, 18]. The pathogenesis of pancreatitis after CSEMS deployment is considered to be occlusion of the pancreatic duct orifice by physical compression of the stent covered section [6], and care should be taken especially when a fully covered type stent is deployed. Thus, in order to ensure pancreatic juice outflow and prevent pancreatitis, EST is generally performed before CSEMS deployment [9–11]. However, when the pancreatic duct is already occluded due to malignant invasion, the need for EST is not clear. Moreover, because EST itself also carries a risk of causing complications, such as bleeding, perforation, and pancreatitis [19], the possibility of EST actually increasing the incidence of complications cannot be ignored. 

There have been a few reports describing EST as not being needed before CSEMS deployment for the prevention of pancreatitis in cases with an occluded pancreatic duct [8, 12–15]. Artifon et al. [12] compared groups with and without EST performed before CSEMS deployment for malignant lower biliary stricture. Although there was no pancreatitis in either group, the group undergoing EST had a higher incidence of complications such as bleeding, perforation, and migration. This report included almost the same number of subjects as our study. However, the incidences of bleeding and perforation in the EST group were higher, at 13.5% (5/37) and 10.8% (4/37), respectively. In our study, the incidence of bleeding in the EST group was only 2.6% (1/38), and there were no perforations. The difference of complication rates may be caused by the method of EST procedure, such as mode setting of the electrosurgical generator and the range and direction of incision. At our hospital, the electrosurgical generator was set for 120 Watt Endocut mode, and the incision range was small or medium. Artifon et al. [12] performed EST with pure cut current. Although our study results support those of previous reports indicating that EST did not reduce the incidence of pancreatitis in the cases of distal malignant obstruction with main pancreatic duct obstruction, EST application significantly increases the incidence of complications. However, procedure time was longer in EST group as compared with that in non-EST group in our result; a potential advantage of the non-EST approach could be a shorter procedure. EST is performed prior to CSEMS deployment to improve the insertability of a medical device into the bile duct for biopsy, brushing cytology, and so forth. We consider that appropriately performed EST appears to be acceptable.

Kawakubo et al. [8] indicated biliary stricture caused by diseases other than pancreatic cancer and a stent with high axial force to be risk factors for pancreatitis after CSEMS deployment based on multivariate analysis but found no association with EST. However, when using partially covered type stent, the pancreatic duct orifice might not have been closed by the stent cover. CSEMSs used in the study by Artifon et al. [12] were all partially covered type stents, and Kawakubo et al. [8] did not mention the type of CSEMS used. Thus, the risk for developing pancreatitis with the use of a fully covered type stent has not yet been examined sufficiently. In the present study, we also assessed the risk of pancreatitis with using fully covered type CSEMSs. The fully- and partially covered type stents were compared in the non-EST group and there was no difference in the incidence of complications including pancreatitis between these 2 types of stents. Furthermore, comparison between the EST and non-EST groups and between the FC and PC groups within the non-EST group also revealed no apparent difference in serum amylase levels before and after CSEMS deployment. These results suggested that, when there is already pancreatic duct obstruction associated with a malignant tumor, the risk of developing pancreatitis is low regardless of whether EST is performed or the fully- and partially covered stents used. 

Moreover, in our results, nonpancreatic cancer was a risk factor for the development of complications after CSEMS deployment by the multivariate analysis. It is thought to be causally related to high incidence of distal migration in patients with ampullary carcinoma. In the patient of ampullary carcinoma, noncovered metallic stent or plastic tube stent may be better than CSEMS.

Finally, our study has some limitations. This was not a prospective study, so selection biases were present with regard to the type of CSEMS used and the EST procedure. The procedure was performed at the discretion of the endoscopists. Because of the retrospective design of our single center study and the limited number of patients, further prospective studies with a large number of patients should be done to confirm our results.

## 5. Conclusions

EST did not reduce the incidence of pancreatitis and level of amylase in the distal malignant obstruction case with main pancreatic duct obstruction, and EST was not identified as a risk factor of all complications after CSEMS deployment. A potential advantage of the non-EST approach could be a shorter procedure. 

## Figures and Tables

**Table 1:** Patient characteristics.

	EST group (*n* = 38)	Non-EST group (*n* = 41)	*P* value
Age (mean ± SD)	72 ± 11	73 ± 9	0.589
Sex (male/female)	21/17	19/22	0.428
Pancreatic cancer	37	36	0.239
Ampullary carcinoma	0	4	0.144
Gallbladder cancer	1	0	0.969
Metastatic pancreatic cancer	0	1	0.969
Incision range of EST			
Small	20		
Medium	18		
Large	0		
Covered self-expandable metal stent			
Fully covered type	9	13	0.427
Partially covered type	29	28	
Stent length (cm, mean ± SD)	6.0 ± 1.0	5.8 ± 1.0	0.380
Procedure time (min, mean ± SD)	34 ± 16	27 ± 12	0.032
Follow-up period (day, mean ± SD)	216 ± 224	157 ± 183	0.202

EST: endoscopic sphincterotomy.

**Table 2:** Comparison of complications between the EST and non-EST groups.

	EST group (*n* = 38)	Non-EST group (*n* = 41)	*P* value
Serum amylase level (IU/L, mean ± SD)			
Before CSEMS placement	130 ± 196	106 ± 100	0.563
3 hours after CSEMS placement	171 ± 232	136 ± 108	0.638
1 day after CSEMS placement	149 ± 182	174 ± 185	0.324
2 days after CSEMS placement	118 ± 142	154 ± 153	0.144
Complications (%)	7 (18.4)	6 (14.6)	0.650
Pancreatitis	1 (2.6)	1 (2.4)	0.508
Cholecystitis	2 (5.3)	2 (4.9)	0.969
Cholangitis	1 (2.6)	0 (0)	0.969
Bleeding	1 (2.6)	0 (0)	0.969
Liver abscess	1 (2.6)	0 (0)	0.969
Migration	1 (2.6)	3 (7.3)	0.663

EST: endoscopic sphincterotomy. CSEMS: covered self-expandable metal stent.

**Table 3:** Comparison of complications between the FC and PC groups within the non-EST group.

	FC group (*n* = 13)	PC group (*n* = 28)	*P* value
Serum amylase level (IU/L, mean ± SD)			
Before CSEMS placement	100 ± 101	108 ± 101	0.441
3 hours after CSEMS placement	141 ± 102	133 ± 112	0.695
1 day after CSEMS placement	201 ± 201	162 ± 180	0.547
2 days after CSEMS placement	135 ± 126	163 ± 165	0.933
Complications (%)	3 (23.1)	3 (10.7)	0.570
Pancreatitis	0 (0)	1 (3.6)	0.691
Cholecystitis	2 (15.4)	0 (0)	0.177
Cholangitis	0 (0)	0 (0)	
Bleeding	0 (0)	0 (0)	
Liver abscess	0 (0)	0 (0)	
Migration	1 (7.7)	2 (7.1)	0.561

FC: fully covered type. PC: partially covered type. EST: endoscopic sphincterotomy.

CSEMS: covered self-expandable metal stent.

**Table 4:** Risk factors for overall complications after CSEMS deployment (univariate analysis).

	Complication (+) (*n* = 13)	Complication (*−*) (*n* = 66)	*P* value	OR (95% CI)
Female gender	7	32	0.770	1.2 (0.38–4.1)
Age (<60 years)	4	4	0.022	6.9 (1.5–33)
Nonpancreatic cancer	4	2	0.006	14 (2.3–89)
Periampullary diverticulum	3	8	0.377	2.2 (0.49–9.6)
Non-EST	6	35	0.765	0.76 (0.23–2.5)
Fully covered type stent	5	17	0.499	1.8 (0.52–6.3)
Pancreatic duct injection	3	17	1.000	0.87 (0.21–3.5)
Biopsy of the bile duct	0	4	1.000	—
Cytology of bile juice	1	5	1.000	1.0 (0.11–9.5)
Intraductal ultrasonography	1	2	0.421	2.7 (0.22–32)
Procedure time (>45 minutes)	2	8	0.666	1.3 (0.2–7.1)

CSEMS: covered self-expandable metal stent. EST: endoscopic sphincterotomy.

**Table 5:** Risk factors for overall complications after CSEMS deployment (multivariate analysis).

	*P* value	OR (95% CI)
Age (<60 years)	0.003	12 (2.2–63)
Nonpancreatic cancer	0.001	24 (3.5–165)

CSEMS: covered self-expandable metal stent.
